# Plasmonic detection of mercury via amalgam formation on surface-immobilized single Au nanorods

**DOI:** 10.1080/14686996.2016.1258293

**Published:** 2017-01-09

**Authors:** Carola Schopf, Alfonso Martín, Daniela Iacopino

**Affiliations:** ^a^ Tyndall National Institute, Nanotechnology Group, University College Cork, Cork, Ireland

**Keywords:** Gold nanorods, mercury detection, plasmonic, darkfield microscopy, amalgam formation, 40 Optical, magnetic and electronic device materials, 105 Low-Dimension (1D/2D) materials, 204 Optics / Optical applications, 208 Sensors and actuators, 212 Surface and interfaces

## Abstract

Au nanorods were used as plasmonic transducers for investigation of mercury detection through a mechanism of amalgam formation at the nanorod surfaces. Marked scattering color transitions and associated blue shifts of the surface plasmon resonance peak wavelengths (*λ*_max_) were measured in individual nanorods by darkfield microscopy upon chemical reduction of Hg(II). Such changes were related to compositional changes occurring as a result of Hg–Au amalgam formation as well as morphological changes in the nanorods’ aspect ratios. The plot of *λ*_max_ shifts vs*.* Hg(II) concentration showed a linear response in the 10–100 nM concentration range. The sensitivity of the system was ascribed to the narrow width of single nanorod scattering spectra, which allowed accurate determination of peak shifts. The system displayed good selectivity as the optical response obtained for mercury was one order of magnitude higher than the response obtained with competitor ions. Analysis of mercury content in river and tap water were also performed and highlighted both the potential and limitation of the developed method for real sensing applications.

## Graphical abstract

**Figure:**
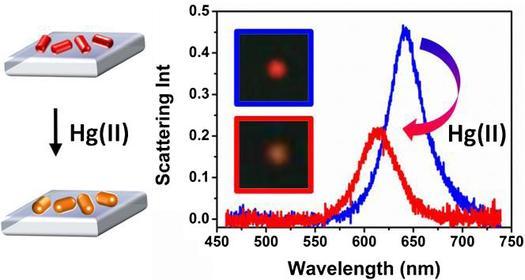

## 1. Introduction

Mercury is a highly toxic heavy metal that is widely dispersed in the environment and can be found in water, air and soil. Mercury is not biodegradable and remains in ecological systems and in the food chain indefinitely, exposing top-level predators and humans potentially to very high levels of pollution.[1–3] Upon extended exposures to mercury (for example from water) humans can be severely harmed and suffer from brain, heart, kidney, lung and immune system damage.[4,5] For this reason large efforts have been devoted to the development of strategies for trace detection of mercury in the environment. Accurate and precise techniques exist for trace mercury analysis (down to nM and pM range) such as gas chromatography inductively coupled plasma mass spectrometry (GC-ICP-MS),[6] atomic fluorescence spectrometry (AFS),[7] inductively coupled plasma atomic emission spectroscopy (ICP-AES) [8] and reversed-phase high performance liquid chromatography (HPLC).[9] In spite of their excellent sensitivity, these techniques are costly, time consuming (requiring pre-concentration steps), and non-portable. Therefore, there is a growing interest in development of alternative simpler and lower cost approaches for on-site analysis, extending current applicability and leading to novel miniaturized sensing solutions.

Noble metal nanoparticles have been proven particularly suitable for detection of mercury due to their sensitive optical response arising from localized surface plasmon resonance (LSPR). Their versatile surface chemistry has been exploited to modify the surface of nanoparticles with a large range of organic and bio molecules, leading to specific interactions with mercury.[10,11] Plasmonic nanoparticles have also been successfully used for label-free detection of mercury based on colorimetric (mercury-induced aggregation) response in solution, with sensitivity down to nM metal concentration detection.[12,13] Among nanoparticle shapes, nanorods have been identified as ideal candidates for plasmon sensing due to their large spectral shift for a given change in refractive index, which exhibits much higher sensitivity compared to that of spherical counterparts.[14–16] For example, sensitive detection of mercury was obtained with Au nanorod solutions spiked with known amounts of Hg(II) in presence of reducing agent NaBH_4_.[17] High optical selectivity and sensitivity was achieved due to chemical reduction of Hg(II) and consequent formation of an amalgam between mercury and gold, which shifted the maximum absorbance wavelength of the nanorods’ longitudinal plasmon mode. Towards development of sensor platforms, more recently the same approach was used to detect mercury with Au nanorods immobilized on glass slides.[18,19] The chemical reduction of Hg(II) was accompanied by a visible color transition of nanorod glass slides from blue to red as a result of amalgam formation. Analytical figures of merit showed precise and accurate detection down to nM level.[18]

However sensitive, a system response based on the collective contribution of many nanorods is inherently broadened by the nanorod size distribution and by the possible presence of small clusters and it is in general affected by the nanorod spatial distribution.[20,21] In contrast, interrogation of mercury response at single particle level results in fewer variables and narrower optical responses and should, potentially, lead to development of highly sensitive sensors. For example, James et al. demonstrated detection of mercury vapors with attogram resolution with single nanorods deposited on a substrate.[22] Monitoring of single nanorods allowed comparison of shape and size effects with distinct measurements rather than statistical characterization. In addition, investigation of the sensing mechanism at the single particle level offers the opportunity to deepen our understanding of mercury adsorption process at the nanoscale, which is mandatory for the design of optimized plasmonic-based mercury sensors suitable for real-sample analysis.

In this paper, we used individual Au nanorods immobilized on solid substrates as plasmonic transducers to investigate the deposition of mercury towards development of a miniaturized mercury plasmonic sensing system. The deposition of mercury on nanorods following chemical reduction of Hg(II) was monitored by measuring the shift of individual nanorods longitudinal LSPR using darkfield microscopy. A marked scattering color change, mostly from intense red to pale orange, was observed in individual nanorods immersed in Hg(II) solutions in presence of reducing agent NaBH_4_. Remarkable blue shifts of nanorod LSPR modes were measured. Exposure to incremental amounts of mercury resulted in a linear response in the 10–100 nM mercury concentration range. The spectroscopic shifts occurred as result of compositional and morphological changes of the nanorods induced by amalgam formation. The sensitivity of the system was ascribed to the narrower full width at half maximum (FWHM) of single nanorod scattering spectra compared to ensemble nanorod spectra, which allowed for accurate determination of peak shifts. Selectivity was tested by taking similar measurements with competitor ions, which produced optical responses one order of magnitude lower than mercury. These results demonstrate the potential suitability of gold nanorods for environmental analysis. However, in the manuscript we also stress the limitation imposed by chemically synthesized nanorods for development of reproducible sensors and we highlight the necessity of protocols for fabrication of reproducible substrates both in terms of nanorod shape/size and nanorod density. Initial investigation on real water matrices showed the high potential of this method for sensitive environmental analysis as well as the need to implement pre-treatment protocols before the presented process can be used for real analytical applications.

## 2. Experimental section

### 2.1. Materials

Tetrachloroauric acid, sodium borohydride, ascorbic acid, silver nitrate, cetyltrimethyl-ammoniumbromide (CTAB), mercury(II) chloride, cadmium perchlorate hydrate, lead(II) chloride, nickel(II) chloride, manganese(II) chloride and copper(II) chloride and (3-aminopropyl)trimethoxysilane (APTES) were obtained from Sigma-Aldrich (Ireland). All glassware was cleaned with piranha solution prior to nanorod synthesis. Milli-Q water (resistivity > 18 MΩ cm^−1^) was used for all the experiments.

### 2.2. **Synthesis of nanorods**

Au nanorods (aspect ratio, AR = 3.0) were synthesized by a seed-mediated method reported by Alvarez-Puebla et al. [23]. The final CTAB concentration in the nanorod water dispersion was maintained between 0.1 and 0.35 mM.

### 2.3. Immobilization of nanorods on glass substrates

Binary alignment marks were etched on microscope slides used as substrates to allow the exact localization of each nanorod. Prior to the nanorod deposition, silanization of glass substrates was performed. Microscope slides were sonicated in acetone and deionized water for 10 min, rinsed with deionized water and dried under a stream of nitrogen. Subsequently, glass microscopes were treated with oxygen plasma of 50 W for 5 min and immersed in a 3% APTES:MeOH solution for 30 min. The glass substrates were rinsed with methanol and deionized water twice to remove excess unbound silane. Crosslinking of the silane was achieved by placing the substrates in a fan operated oven at 120 °C. Immobilization of Au nanorods was performed by depositing a droplet (100 μl) of nanorod aqueous solution on the substrate for 3 min followed by rinsing with 40 °C deionized water to remove excess surfactant.

### 2.4. Detection of mercury

HgCl_2_ was dissolved in deionized water to prepare Hg(II) stock solutions with concentrations of 100 μM and 10 μM. Excess (0.01 M) of NaBH_4_ was employed for the chemical reduction of Hg(II). Nanorods immobilized on glass substrates with were firstly immersed in 10 ml 0.01 M NaBH_4_ for 10 min followed by immersion in NaBH_4_ solutions (10 ml, 0.01 M, 10 min) to which aliquots from HgCl_2_ stock solutions were added to produce a desired Hg(II) concentration. Darkfield images and spectra were then acquired on exposed nanorod substrates. The procedure was repeated for solutions with increased Hg(II) concentration.

For real water analysis, a sample of river water was taken from the river Lee outside the institute and a sample of tap water was taken from a tap in the institute. One hundred ml of each sample was externally analyzed by ICP-MS for metal contents by a certified testing service (Fitz Scientific, Ireland). For mercury detection measurements the river and tap water samples were filtered (0.45 μm pore diameter) and spiked with HgCl_2_ to produce concentrations between 5 and 100 nM.

### 2.5. Optical characterization

**Figure 1.**
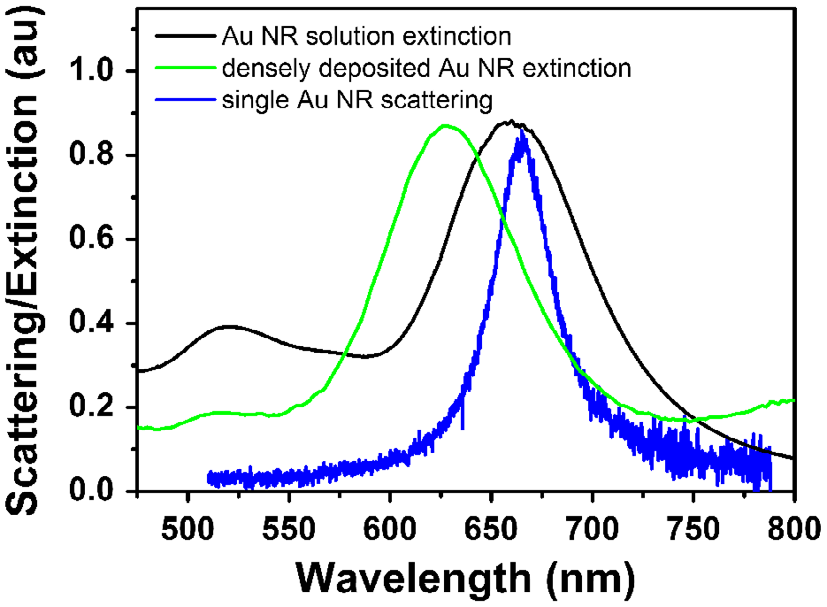
Extinction spectra of Au nanorods (NRs) dispersed in aqueous solution (black curve) and deposited on glass substrate (green curve); scattering spectrum of single Au nanorod deposited on glass substrate (blue curve).

The UV-vis spectrum of nanorod aqueous solution (Figure 1) was acquired with an Agilent/HP 8453 UV-vis spectrophotometer (200 nm < λ < 1100 nm, Dublin, Ireland). Extinction spectrum of gold nanorods deposited on glass coverslips (Figure 1) was acquired with an inverted IX-71 Olympus microscope under halogen lamp (100 W) illumination. The spectrum was acquired by directing the light collected by the objective into the entrance of slit of a monochromator (SP-300i, Acton Research, UK) equipped with a thermoelectrically cooled, back illuminated CCD (Spec10:100B, Princeton Instruments, UK).

### 2.6. Darkfield microscopy

The same inverted IX-71 Olympus microscope and monochromator (SP-2356, Acton Research) were used for acquisition of darkfield spectra of individual Au nanorods in combination with an oil immersion darkfield condenser (Olympus U-DCW) and a 100× objective (Olympus MPlanApo 100x/0.95 NA). Typical spectra acquisition time was 60 s. In order to obtain scattering spectra from individual nanorods obtained spectra were subtracted and divided by a background scattering spectrum taken from a nearby clean area on the sample. The spectra were fitted with a Lorentzian function to determine the peak scattering wavelength.

### 2.7. Electron microscopy

A field emission SEM (JSM-6700F, JEOL UK Ltd) operating at a beam voltage of 10 kV was used to acquire images of individual Au nanostructures.

## 3. Results and discussion

Au nanorods were synthesized according to an overgrowth method reported by Alvarez-Puebla et al. [23]. The method produced Au nanorods of average size 21(±4) × 61(±6) nm. The extinction spectrum of nanorods dispersed in aqueous solution presented characteristic transversal and longitudinal plasmon peaks centered at 520 nm and 660 nm, respectively (see Figure 1). The FWHM of the longitudinal peak was calculated to be 78 nm. For large ensembles of nanorods deposited on glass substrates the transversal and longitudinal plasmon peak positions shifted to 515 nm and 631 nm, respectively. The longitudinal peak FWHM slightly decreased to 72 nm. In comparison, the scattering spectrum of a single nanorod in air measured by darkfield spectroscopy showed only the plasmon peak associated to the longitudinal mode. The spectrum was centered at 666 nm and exhibited a FWHM of 37 nm, narrower compared to the spectra of nanorod ensembles which were broadened by a distribution of shape and size.

**Figure 2.**
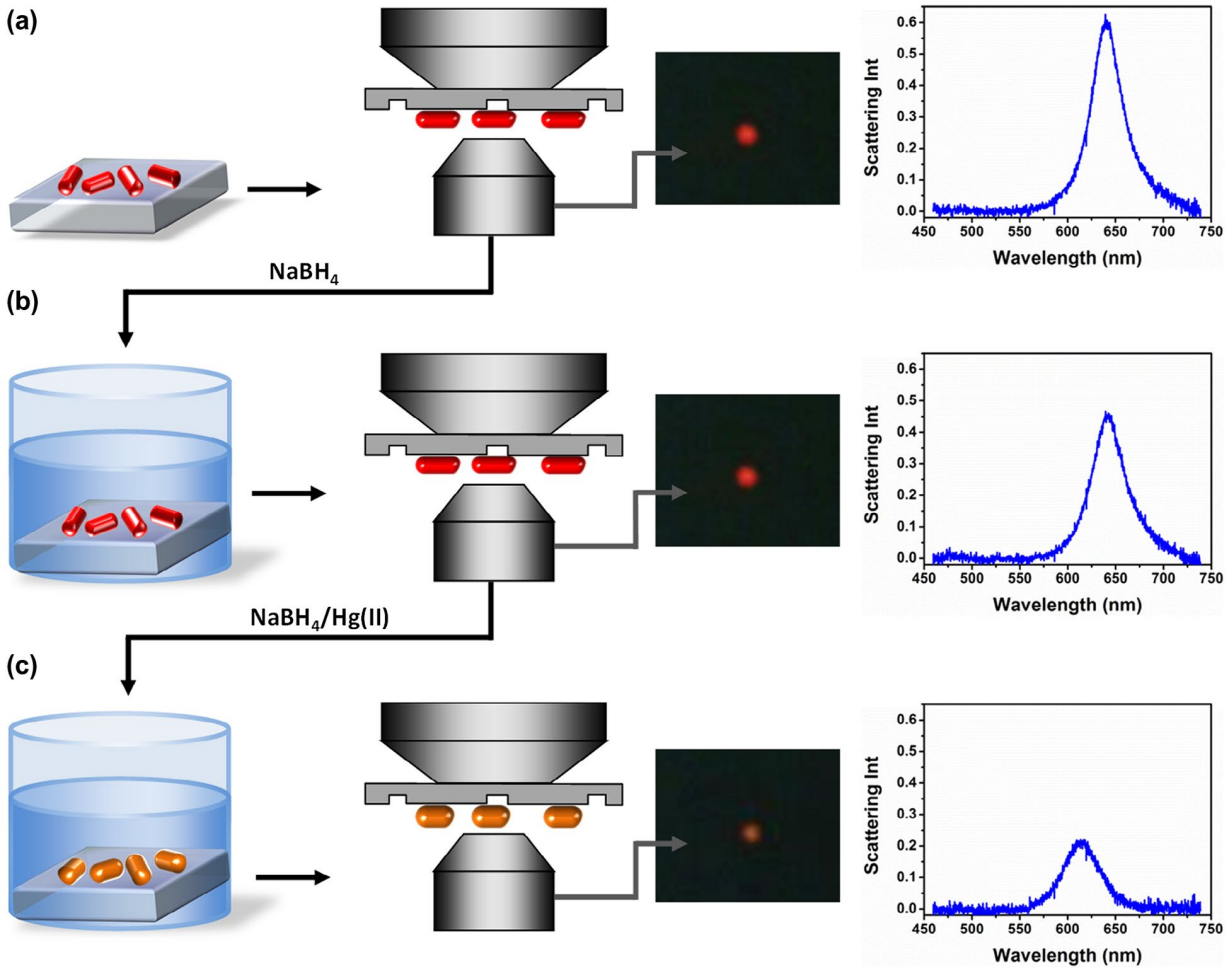
Schematic of Au nanorod amalgamation detection with substrate-immobilized nanorods: Darkfield microscopy is firstly performed on Au nanorod substrates and a scattering spectrum is recorded of selected nanorod (a); Substrates are immersed in NaBH_4_ solution and the spectrum of the same selected nanorod is recorded by darkfield spectroscopy (b); Substrates are immersed in HgCl_2_/NaBH_4_ solution and the nanorod spectrum is recorded again by darkfield spectroscopy (c).

Figure 2 shows the procedure used for mercury detection using nanorod-immobilized glass substrates. Specifically, the nanorod-immobilized substrate was placed in an optical microscope and darkfield images and scattering spectra of selected nanorods were collected (a). The glass slide with Au nanorods was subsequently immersed in an aqueous solution of NaBH_4_ and placed again in the optical microscope where darkfield images and scattering spectra of the same selected nanorods were collected (b). This step was performed in order to ensure that exposure to the reducing agent NaBH_4_ did not generate any optical response of the nanorods. Subsequently, the nanorod substrate was immersed in aqueous solutions of NaBH_4_ containing aliquots of Hg(II) solution. Darkfield images and scattering spectra of the same selected nanorods were collected following Hg(II) reduction (c). This last step was repeated with solutions of increasing Hg(II) concentration. As a representative example, Figure 2 also shows darkfield images and scattering spectra of a single nanorod before and after nanorod immersion in a 100 nM Hg(II) solution. The selected nanorod displayed intense red scattering color typical of elongated particles and was characterized by a scattering intensity peak centered at 640 nm with an FWHM of 41 nm (a). After immersion in NaBH_4_ no appreciable changes in the scattering color or in the scattering spectrum of the selected nanorod were detected (b). In contrast, after addition of HgCl_2_ to the solution and its consequent reduction by NaBH_4_, the selected nanorod scattering color became orange. Accordingly, the corresponding scattering spectrum λ_max_ blue shifted to 610 nm and its FWHM increased to 50 nm (c).

The optical changes occurring on Au nanorods were associated to the reduction of mercury and formation of an Au–Hg amalgam. The process of mercury reduction and its deposition on substrate-immobilized nanorods was investigated in details in a previous publication.[24] A correlated dark-field/electron microscopy approach was used and revealed that the nanorod optical blue shifts observed upon chemical reduction of Hg(II) were associated to deposition of mercury on gold surfaces and subsequent reduction of nanorods’ aspect ratio towards spherical shapes. Therefore, the optical changes described in Figure 2 were ascribed to the occurrence of two concomitant effects: alteration of the nanorod surface composition due to deposition of mercury and transition from elongated to spherical shape.

Prior to exposure to Hg(II) solutions of varying concentration, the effect of NaBH_4_ and Hg(II) exposure on the optical properties of immobilized Au nanorod was investigated. Nanorods were immersed in NaBH_4_ solutions and the optical changes were monitored over time. Nanorod scattering spectra displayed a small λ_max_ red shift (see Figure S1 in supporting information) in the first 10 min followed by no further optical changes. Similarly, exposure to HgCl_2_ solutions led to a red shift that reached a plateau after 10 min of monitoring. For this reason in all darkfield measurements substrates were immersed in NaBH_4_/HgCl_2_ solutions for 10 min before spectral examination.

**Figure 3.**
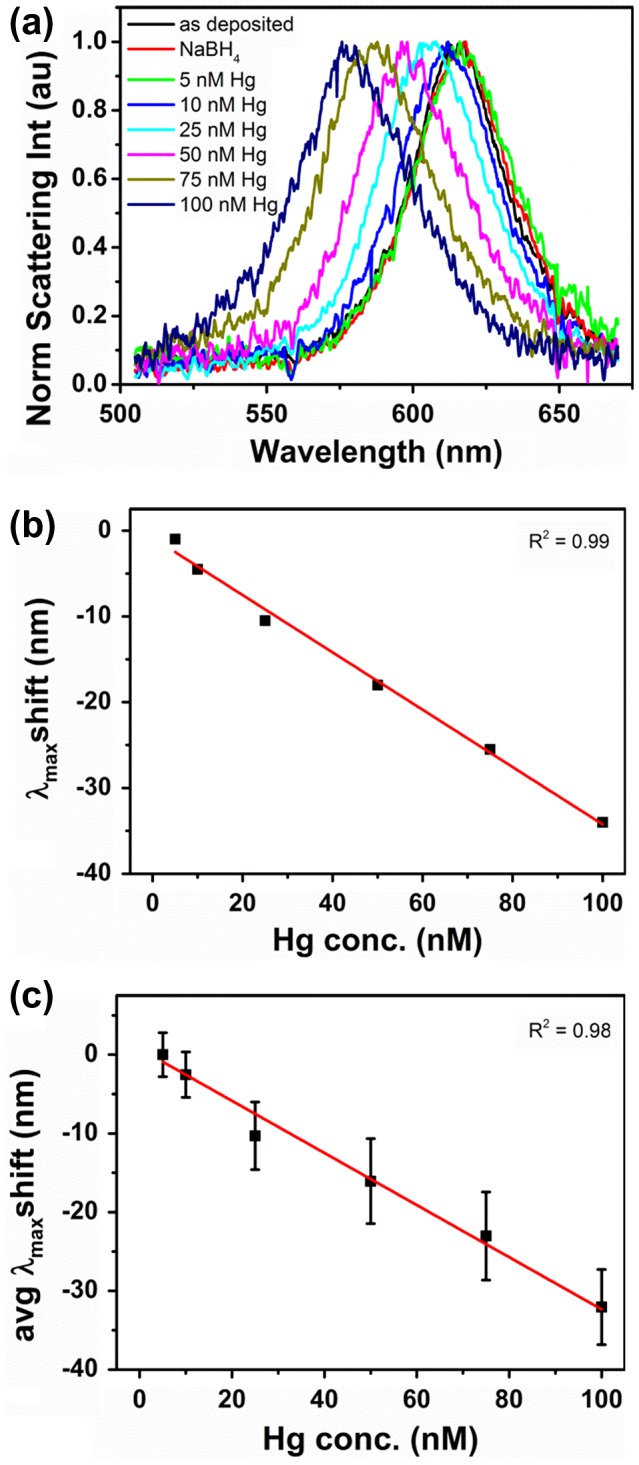
(a) Normalized scattering spectra of an individual Au nanorod exposed to incremental amounts of Hg(II) in presence of reducing agent NaBH_4_; (b) linear dependence of single nanorod spectrum maxima on Hg(II) concentration; (c) linear dependence of single nanorod spectrum maxima on Hg(II) concentration averaged over 12 nanorods.

Figure 3(a) shows normalized scattering spectra of a single Au nanorod exposed to increasing amounts of Hg(II). Prior to Hg(II) addition, spectra of the as-deposited nanorod and the nanorod exposed to NaBH_4_ were also recorded. The close similarity of the corresponding black and red curves excluded any contribution from the reducing agent during Hg(II) exposure. All other spectra were recorded after 10 min immersion in Hg(II) containing solutions, when the optical response of nanorods was stable and further spectral changes due to addition of chemicals were not observed.

Upon exposure to increasingly concentrated Hg(II) solutions, a progressive blue shift of the nanorod scattering peak maximum intensity, λ_max_, was observed. In particular, λ_max_ moved from 616 nm to 612, 606, 599, 591 and 582 nm as the Hg(II) concentration increased from 5 nM to 10, 25, 50, 75 and 100 nM, respectively. The observed spectral changes and the color transition towards the green correlated well with the combined effects of the nanorods’ compositional change and the decrease in aspect ratio.[24–26]

Closer observation also revealed a slight increase in the FWHM of scattering spectra obtained at higher Hg(II) concentrations as expected for increased plasmon damping in mixed composition particles.[27,28]

Figure 3(b) reports the λ_max_ shift of a single Au nanorod in dependence of Hg(II) concentration. λ_max_ values were extracted from Lorentzian fits to the recorded nanorod scattering spectra. A linear response with an *R* ^2^ = 0.99 was obtained for measurements performed in the 5 nM to 100 nM Hg(II) concentration range, demonstrating the potential of this system for sensitive environmental analysis. The exposure of Au nanorods to 10 nM Hg(II) solution resulted in a –4.0 nm shift, above the spectral resolution of 2 nm, suggesting that slightly lower concentrations could be detected by the system. For comparison, Figure 3(c) reports the λ_max_ shift averaged over 12 Au nanorods measured on the same substrate as a function of Hg(II) concentration. A linear response with an *R* ^2^ = 0.98 was still obtained for measurements performed in the 5–100 nM Hg(II) concentration range. However, relatively high standard deviation values were associated to the measurements, which led to an estimated limit of detection (LOD) of only 45 nM. The LOD was estimated as LOD = 3·*stdev/m* with *stdev* being the maximum standard deviation obtained for the data points in Figure 3(c), and m being the slope of the linear fit to the data points. The high standard deviation values arose from nanorod size and shape distributions obtained by chemical synthesis which in turn led to a relatively large distribution of nanorod sensitivities. The impact of nanorod size and shape distribution on their sensitivity towards mercury vapor was also reported by James et al. [22] who observed that larger size nanorods were more sensitive to mercury than smaller size nanorods. This further explains the poor LOD achieved when a higher number of nanorods on a substrate was probed (Figure 3(c)) and highlights the difficulty of making a reliable sensor even at single particle level based on chemically synthesized nanorods, as the same size/shape nanorod should always be probed in order to obtain a reproducible response. James et al. also reported that the density of nanoparticles in a film affected the mass limit of detection.[29] They observed that λ_max_ shifts depended on the amount of mercury adsorbed per particle which was found to be directly proportional to the surface density of nanoparticles in the film. That means that for sensitive and reproducible responses a deposition method able to deliver a controlled number of nanorods on the substrate should be used in order to ensure reproducibility of results. The fabrication of substrates with a known number of deposited nanorods of homogeneous size (achievable by lithography processes) would eliminate potential sensitivity variations due to differences in nanoparticle density and shape distribution on the substrate. Alternatively, normalizing the λ_max_ shift as function of [Hg]/number of nanorod could be effective in addressing these issues. The use of dedicated spectroscopic imaging tools (e.g. hyperspectral imaging) for collection of individual spectroscopic responses from a large number of nanorods could also be considered.[30] However, in both cases loss of sensitivity might occur due to the probing of multiple nanorods.

**Figure 4.**
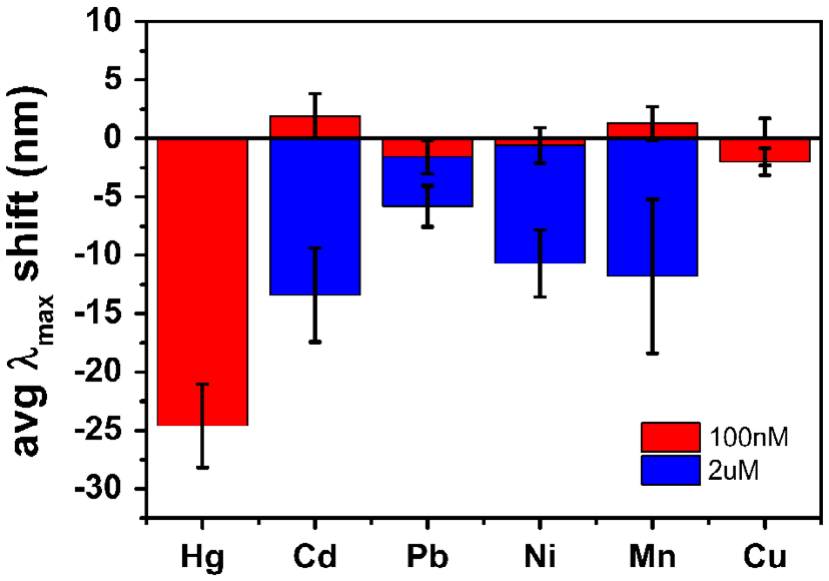
Average scattering maxima wavelength shifts of Au nanorods exposed to 100 nM HgCl_2_, and 100 nM or 2 μM of Cd(ClO_4_)_2_, PbCl_2_, NiCl_2_, MnCl_2_, and CuCl_2_.

In order to investigate the specificity of the mercury amalgamation process, Au nanorods were exposed to 100 nM solutions of HgCl_2_, Cd(ClO_4_)_2_, PbCl_2_, NiCl_2_, MnCl_2_ or CuCl_2_ in the presence of 0.01 M NaBH_4_. Results are shown in Figure 4 for a Hg(II) concentration of 100 nM and competitive ion concentrations of 100 nM (red columns) and 2 μM (blue columns), respectively. Each column represents the λ_max_ shift averaged over 10 measured nanorods analyzed after 10 min exposure to the investigated ion. At a 100 nM concentration of Pb(II), Ni(II) and Cu(II) the nanorods’ LSPR showed a small blue shift below 3 nm. 100 nM Cd(II) and Mn(II) solutions caused a LSPR red shift also below 3 nm. In contrast, a 100 nM Hg(II) solution caused a strong LSPR blue shift of 25 nm. When the competitor ion concentration was increased by 20 times, all ions showed blue shifts between 7 and 12 nm, still reasonably small compared to the lower concentration Hg(II) response. The specificity of Hg(II) detection could be attributed to the high standard reduction potential of Hg(II) (+ 0.851V) compared to the other M(II) ions (Cd(II) = −0.403V; Pb(II) = −0.126V; Ni(II) = −0.257V; Mn(II) = −1.185V; Cu(II) = +0.342V) [31]. However, as the tests were carried out in several solutions, it is likely that some reduction of metal species occurred during addition of NaBH_4_. However, the lower cohesive energy of Hg (0.67 eV) compared to other metals, such as Cd (1.16 eV), Pb (2.03 eV), Ni (4.44 eV), Mn (2.92 eV) and Cu (3.49 eV) [32] facilitated inter-diffusion of Hg and Au compared to the other metals, where no changes associated to nanorod morphology were expected.

**Figure 5.**
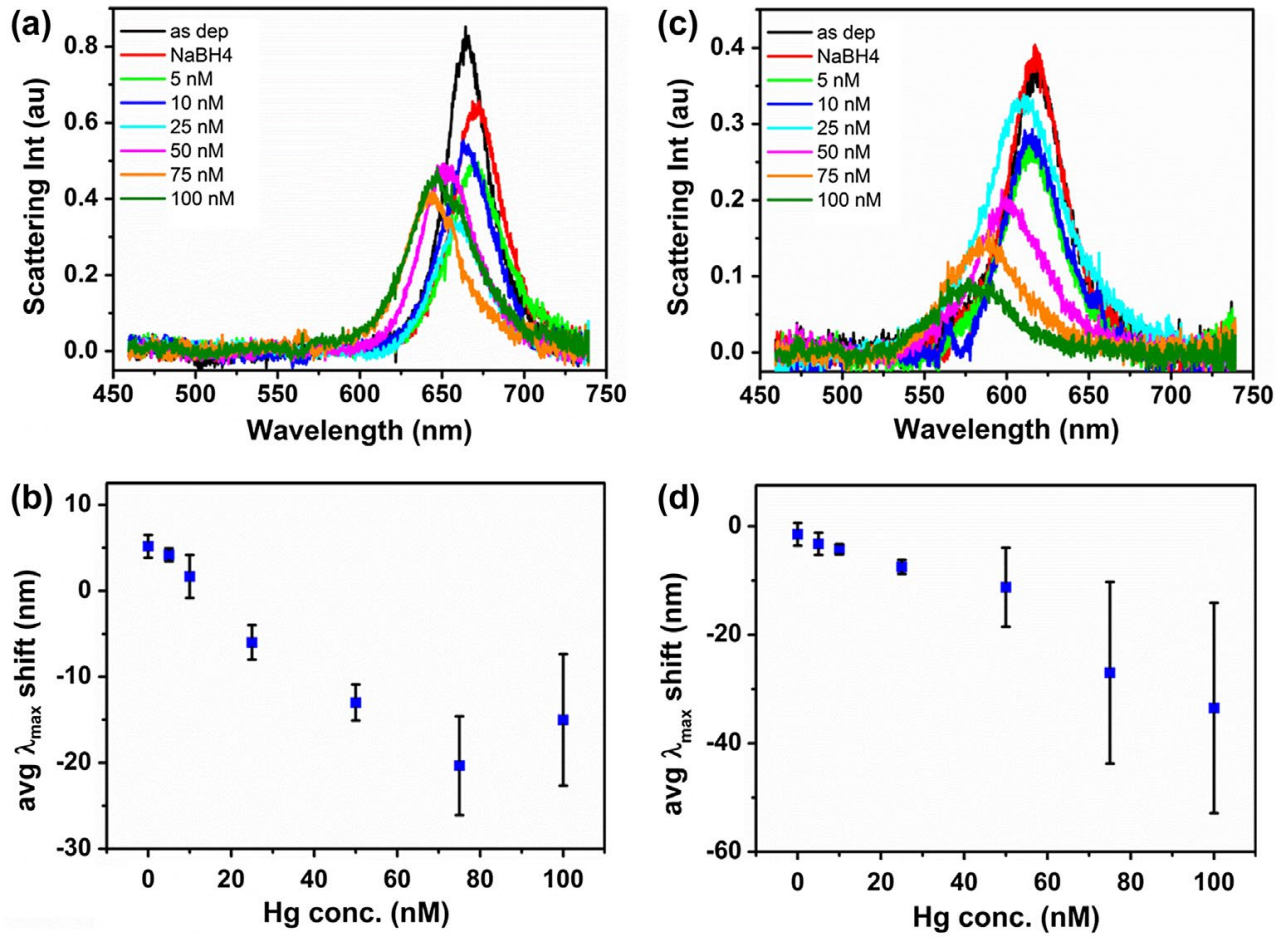
Typical set of scattering spectra for an individual gold nanorod exposed to incremental amounts of Hg(II) in presence of reducing agent NaBH_4_ in river water (a) and tap water (c). Average scattering maxima wavelength shifts of gold nanorods in dependence of Hg(II) concentration averaged over 10 nanorods on one sample in river water (b) and tap water (d).

Finally, the detection of Hg(II) ions was tested in real water samples in order to assess its efficacy in complex matrices. A sample of river water was taken from the river outside the institute and a sample of tap water was taken from a tap in the institute. Parts of the samples were externally analyzed by ICP-MS for metal contents by a certified testing service. Details of river and tap water compositions are in Table S1, supporting information. The river and tap water were filtered and spiked with HgCl_2_ in a concentration range of 5–100 nM for measurements. Figure 5 shows the response of Au nanorods towards Hg(II) in river and tap water. The single nanorod spectra in Figure 5(a) show a shift of the peak scattering wavelength λ_max_ from 665 nm to 672 nm after immersion of the sample in 0.01 M NaBH_4_ river water. Following this red shift, after treating the sample with Hg(II) containing solutions λ_max_ gradually blue shifted from 672 nm to 671, 666, 661, 654 and 644 nm as the concentration increased from 5 nM to 75 nM Hg(II). For 100 nM Hg(II) a red shift to 649 nm was observed. The initial red shift after immersion in 0.01 M NaBH_4_ river water was observed for all 10 analyzed single nanorods (see average shift in Figure 5(b)) and also in a repeat experiment (data not shown). As this was not observed in the experiments carried out in deionized water, this red shift was ascribed to deposition of some component present in the river water.

The red shift could have been caused by the deposition of one of the metals present in higher concentration like Al, Fe or Mn, but also organic components that might be present in the river water. Whatever the cause, the nanorods were evidently still accessible to reduced mercury in solution as the nanorods scattering spectrum gradually blue shifted with increasing mercury content in the solution. Compared to the peak position in NaBH_4_ solution small blue shifts were observed for 5 and 10 nM mercury concentrations. However, only for concentrations above 10 nM a blue shift compared to the as-deposited nanorod was registered. The red shift of the nanorods’ scattering spectrum observed at 100 nM Hg(II) concentration was caused by the appearance of a deposit on the sample. The origin of the deposit was not investigated in close detail. However, it could be removed by gentle rinsing with ethanol, indicating its possible organic nature.

As a second real water example, mercury detection in tap water was investigated. Figure 5(c) shows the response of Au nanorods towards HgCl_2_ in tap water. The as-deposited nanorod’s peak scattering wavelength λ_max_ of 618 nm remained unchanged after immersion in 0.01 M NaBH_4_ tap water, then gradually blue shifted to 616, 616, 611, 602, 588 and finally 580 nm as the concentration increased from 5 nM to 100 nM HgCl_2_. Contrary to the measurements in river water, in tap water no red shift was observed after immersion of the sample in 0.01 M NaBH_4_ solution without HgCl_2_. Consequently, any λ_max_ shifts observed were attributed to the effect of mercury. Unfortunately, with the increase of mercury concentration the same deposit as observed in the river water experiments appeared on the sample. As the deposit did not cover all areas of the sample homogeneously diverging responses of nanorods at different locations on the sample were obtained, responsible for the large error bars in the average λ_max_ shifts for 50, 75 and 100 nM HgCl_2_ concentrations in Figure 5(d).

## 4. Conclusions

In conclusion, we showed that individual Au nanorods immobilized on substrates displayed a strong spectroscopic response when exposed to chemically reduced mercury. The response was ascribed to formation of a Hg–Au amalgam which caused alteration in nanorod shape and composition. This constituted the basis for demonstration of highly sensitive and selective sensing based on single nanorods. In fact, appreciable λ_max_ shifts were obtained for Hg(II) concentrations as low as 10 nM and the λ_max_ shift response was linear within Hg(II) concentration range 10–100 nM. Our investigation of single nanorod performance compared to ensemble performance showed that a gap still exists between laboratory use demonstration and applicability to real samples. In particular, for the presented system, issues related to nanostructure distribution will have to be addressed and pre-purification protocols should be implemented before real-world analysis can be reliably performed. Nevertheless, the reported linear correlation and high selectivity make this approach potentially suitable for on-site analysis using a miniaturized portable spectrometer.

## Disclosure statement

No potential conflict of interest was reported by the authors.

## Funding

This work was supported by the European Commission under the FP7 NMP projects ‘Hysens’ [263091] and ‘Rosfen’ [312829], and the Irish HEA PRTLI program [Cycle 3 ‘Nanoscience’ and Cycle 4 ‘Inspire’].

## Supplemental data

Supplemental data for this article can be accessed here. [http://dx.doi.org/10.1080/14686996.2016.1258293]

## Supplementary Material

Supporting_Information.docx
