# Supplementary material for: Plasmonic detection of mercury via amalgam formation on surface-immobilized single Au nanorods
Source: Sci Technol Adv Mater. 2017 Jan 9;18(1):60–7. doi: 10.1080/14686996.2016.1258293 (PMC5256255; doi:10.1080/14686996.2016.1258293)
Supplement: Supporting_Information.docx [file tsta_a_1258293_sm4076.docx]

Supporting Information

Plasmonic Detection of Mercury *via* Amalgam Formation on Surface-Immobilized Single Au Nanorods

Carola Schopf, Alfonso Martín, Daniela Iacopino*

Tyndall National Institute, University College Cork, Lee Maltings, Dyke Parade Cork, Ireland

Corresponding Author

*Daniela Iacopino

Tyndall National Institute, University College Cork

Lee Maltings, Dyke Parade, Cork, Ireland

Email: daniela.iacopino@tyndall.ie

**Influence of NaBH_4_ and Hg(II) exposure on Au nanorod optical properties**

The effect of exposure to NaBH_4_ was investigated in order to exclude any contribution from the reducing agent during mercury exposure. The λ_max_ shift of the nanorod scattering band was measured against time and averaged over 10 different nanorods (see Figure S1, black squares). Initially (2 min) a red shift occurred, followed by a blue shift back to the initial wavelength. After 5 min small blue shift of the λ_max_ were measured until after 20 min no further spectral changes were detected. Therefore nanorod samples were immersed for 10 min in reducing agent solution prior to addition of mercury aliquot. In a similar manner the effect of immersion time on the spectral response of nanorods exposed to mercury was tested by adding 100 nM HgCl_2_ and monitoring the spectral changes of 10 nanorods over time (see Figure S1, red circles). A strong blue shift occurred within the first 5 min after which the λ_max_ reached a plateau and no further spectral changes were detected after 10 min. Therefore in all darkfield microscopy experiments substrates were immersed in NaBH_4_/HgCl_2_ solutions for 10 min before spectral examination.





**Figure S1**. λ_max_ shift of nanorod scattering band over time averaged over 10 nanorods immersed in NaBH_4_ (10 mM, H_2_O, black squares) and after addition of 100 nM HgCl_2_

**Table 1**. Atomic content of tap and river water analyzed by ICP-MS

|  | River water content (nM) | Tap water content (nM) |
| --- | --- | --- |
| Al | 1520 | 719.74 |
| Cd | <0.09 | <0.8 nM |
| Cu | 15.53 nM | 1.66 |
| Fe | 291.33 | 167.17 |
| Pb | <0.01 | <1.83 |
| Mn | 189.49 | 184.39 |
| Hg | <0.15 | <0.20 |
| Ni | <4.60 | 11.04 |

**EDX analysis**

EDX elemental mapping and line scans were attempted. However, due to the large interaction volume of electrons with the material and the small volume of a single amalgamated nanostructure, we assume the EDX signal at any location on the nanostructure contains information about the whole structure. Additionally, due to the small volume of the nanostructure, the signal to noise ratio is very low and no conclusive information could be retrieved from the measurements. An example of a recorded line scan is presented in Figure S2.

**Figure S2**. SEM images of gold nanorods a) before and b) after contact with Hg showing the analysed nanorod on the right. EDX line scan of a single gold nanorod-mercury amalgam: c) position of line scan also seen in b), d) Au and Hg elemental counts along the line scan.

**SEM imaging and size analysis of nanorods exposed to Hg(II) solutions**

The shape evolution of Au nanorods exposed to Hg was monitored with SEM imaging in the concentration range from 50 nM to 10 μM. For each sample a statistical analysis of the present nanorod dimensions was performed on 300 nanorods. Figure S3 shows representative SEM images and nanorod length and width histograms for all analyzed samples. At 500 nM Hg(II) concentration coalescence of nanorods was observed until at 10 μM Hg(II) concentration complete spherical reshaping was observed for all deposited nanorods. The histograms of nanorod dimensions for every Hg(II) exposure show the shape evolution from AR = 2.9 to AR = 1.1.


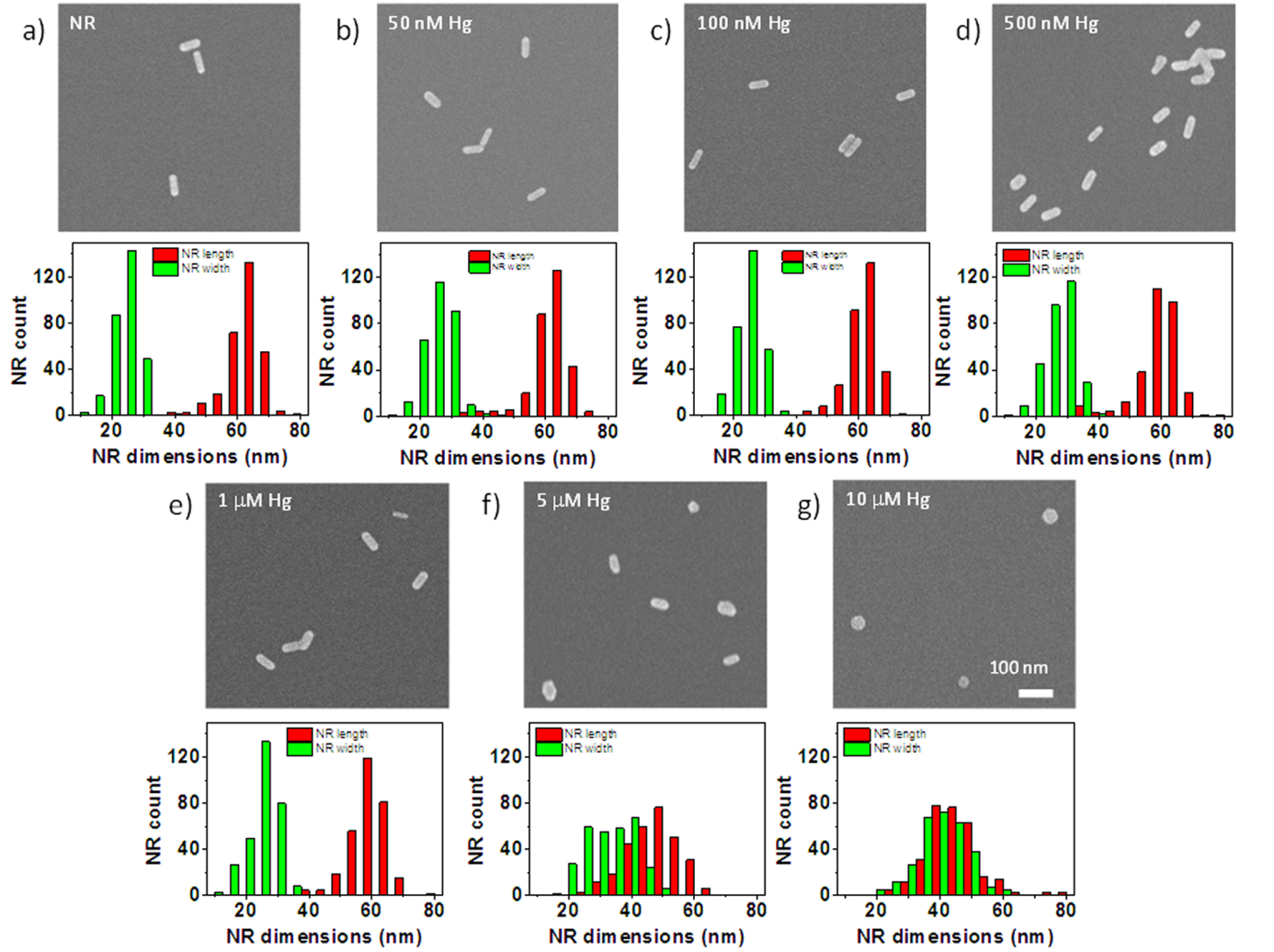


**Figure S3**. SEM images of nanorods a) as deposited and after immersion in Hg(II) solutions b) 50 nM, c) 100nM, d) 500 nM, e) 1 μM, f) 5 μM and g) 10 μM Hg(II). Corresponding length (red) and width (green) analysis averaged over 300 nanorods.

Correlated measurements were taken of Au nanords deposited on a TEM carbon grid before and after exposure to 0.01 M NaBH_4_, 5 μM Hg(II) solutions. As can be seen this Hg concentration is accompanied with nanorods morphological transition to spherical shapes (Figure S4).

**Figure S4**. SEM images of Au nanorods deposited on TEM carbon grids before (a) and after (b) exposure to 5 μM Hg(II) solution.
